# Initiating droxidopa for neurogenic orthostatic hypotension in a patient with Parkinson disease

**DOI:** 10.1007/s10286-017-0429-3

**Published:** 2017-06-19

**Authors:** Daniel Claassen, Mark Lew

**Affiliations:** 1 Department of Neurology, Vanderbilt University, Nashville, TN USA; 2 Department of Neurology, University of Southern California, Los Angeles, CA USA

**Keywords:** Droxidopa, Neurogenic orthostatic hypotension, Parkinson’s disease

## Challenge questions

What is the rationale for choosing pharmacologic therapy for patients with neurogenic orthostatic hypotension? What are the criteria to start with droxidopa?

## Case presentation

Mr. F is a 64-year-old male with Parkinson disease (PD) who presents with fatigue, urinary urgency and frequency, and, more recently, difficulty working as a part-time tollbooth operator. His motor symptoms related to PD have been present for about 7 years, and he has been maintained on carbidopa/levodopa 25 mg/100 mg three times daily (TID) and ropinirole 3 mg TID until now. For the last 6 months, the patient reports a “wobbly or dizzy sensation when standing.” Four months ago, he was seen by his family physician who noted that he had a blood pressure (BP) drop upon standing. The family physician instructed the patient to liberalize fluid and salt intake and gave the patient a prescription for knee-high compression garments.

As part of his current routine, the patient takes a brief nap in the afternoon for 45 min after his lunch when he is off from his job and at home. At work, he reports that he has become more fatigued and that colleagues have noticed that he processes thoughts more slowly. In fact, the patient reports mild memory problems, which became more marked when his family physician prescribed him the anticholinergic drug tolterodine for urinary frequency and urgency.

At a visit to his movement disorder neurologist 3 months ago, his BP was 98/75 mmHg sitting and 75/60 mmHg upon standing with little increase in heart rate (HR). He reported feeling lightheaded and about to faint upon standing. He also reported fatigue, urinary symptoms, and memory complaints, all common in patients with PD and neurogenic OH (nOH). At this visit, his medications were reviewed and adjusted. Treatment of his urinary symptoms with an anticholinergic medication most likely exacerbated his memory problems and daytime sleepiness. Therefore, tolterodine was discontinued. Because dopamine agonists have been associated with low BP, dopamine agonists were decreased to pramipexole dihydrochloride 1 mg at bedtime.

A few weeks after visiting his neurologist, he was placed on medical disability and stopped going to work. His daytime naps increased to twice daily, and he continued to complain about feeling “lightheaded” in the morning and upon standing.

At his next visit to his neurologist, the patient reported that his napping has become more frequent, and he continued to have burdensome symptoms of nOH, which did not remit after his dopaminergic medications were reduced.

## Expert commentary (Dr. Claassen)

Neurogenic OH is a manifestation of autonomic failure, which is characterized by inappropriate norepinephrine (NE) release from sympathetic post-ganglionic nerves when standing. Starting this patient on droxidopa, which is a synthetic precursor of NE [1, 4], is a reasonable option at this stage. Additionally, given that this patient needs to nap several times daily, there is a mechanistic hypothesis that increasing NE levels in the CNS may potentially improve other non-motor symptoms in PD [2], although no large studies have been performed to confirm this.

## Case continuation

The patient was placed on droxidopa 100 mg on a modified TID schedule (doses taken before arising from bed in the morning, at lunchtime, and at least 3–4 h before bedtime) and instructed to increase the dose by 100 mg on this modified TID schedule every 24–48 h until remission of nOH symptoms or up to a maximum droxidopa dosage of 600 mg TID. To make sure that the patient did not develop supine hypertension (sHTN), which is a frequent finding in patients with nOH [3, 5], he was instructed to monitor his BP in the supine and standing (3 min after) positions first thing in the morning during the titration of droxidopa. The patient was also counseled to avoid the supine position during the daytime, to take naps in a recliner chair, and to elevate the head of his bed by 30°–45° at bedtime.

After achieving a droxidopa dose of 500 mg on the modified TID schedule, the patient reported that his nOH symptoms had resolved. This patient has continued this dosage of droxidopa for 6 months and has experienced many fewer nOH symptoms with no adverse effects to date.

## Expert commentary (Dr. Lew)

It is important to carefully consider the risk of sHTN in patients taking medications to increase BP, including droxidopa, midodrine, or fludrocortisone. In this regard, this patient is particularly complicated because he naps several times daily. He needed to be reminded to elevate the head of his bed at least 30°–45° at bedtime, to take his naps in a recliner chair, and to attempt to dose his droxidopa after awakening from his naps, if possible. Additionally, careful review of concomitant medications prescribed by other specialists is crucial. In this case, tolterodine, an anticholinergic agent to treat bladder dysfunction, likely caused and/or contributed to the cognitive impairment.
